# Crystal structure of di­aqua­bis­(7-di­ethyl­amino-3-formyl-2-oxo-2*H*-chromen-4-olato-κ^2^ *O* ^3^,*O* ^4^)zinc(II) dimethyl sulfoxide disolvate

**DOI:** 10.1107/S2056989016009853

**Published:** 2016-06-24

**Authors:** Aaron B. Davis, Frank R. Fronczek, Karl J. Wallace

**Affiliations:** a Department of Chemistry and Biochemistry, University of Southern Mississippi, Hattiesburg, Mississippi 39406, USA; b Department of Chemistry, Louisiana State University, Baton Rouge, Louisiana 70803, USA

**Keywords:** crystal structure, zinc complex, coumarin ligands, hydrogen bonding, DMSO solvate

## Abstract

The structure of the title coordination complex, [Zn(C_14_H_14_NO_4_)_2_(H_2_O)_2_]·2C_2_H_6_OS, shows that the Zn^II^ cation adopts an octa­hedral geometry and lies on an inversion center. Two organic ligands occupy the equatorial positions of the coordination sphere, forming a chelate ring motif *via* the O atom on the formyl group and another O atom of the carbonyl group (a pseudo-β-diketone motif). Two water mol­ecules occupy the remaining coordination sites of the Zn^II^ cation in the axial positions. The water mol­ecules are each hydrogen bonded to a single dimethyl sulfoxide mol­ecule that has been entrapped in the crystal lattice.

## Abstract 2

A near-perfect octa­hedral zinc(II) complex coordinated to two coumarin fluoro­phores.

## Chemical context   

Fluorescent mol­ecular probes have been utilized in the monitoring of anions, cations, and neutral species in many applications in supra­molecular analytical chemistry (Lee *et al.*, 2015 ▸). In particular, derivatives of 1,2-benzopyrone (commonly known as coumarin) have been used extensively as fluorescent chemosensors for a wide range of applications due to their unusual photo-physical properties in different solvent systems and using theoretical calculations (Lanke & Sekar, 2015 ▸; Liu *et al.*, 2013 ▸). There is a plethora of coumarin dyes and their derivatives that have been used as colorimetric and fluorescent sensors (Lin *et al.*, 2008 ▸; Ray *et al.*, 2010 ▸). In fact our own group has used a coumarin–enamine organic compound as a chemosensor for the detection of cyanide ions, *via* a Michael addition approach (Davis *et al.*, 2014 ▸). Additionally, we have utilized a small family of the coumarin chemosensors to discriminate metal ions as their chloride salts utilizing Linear Discriminant Analysis (Mallet *et al.*, 2015 ▸).
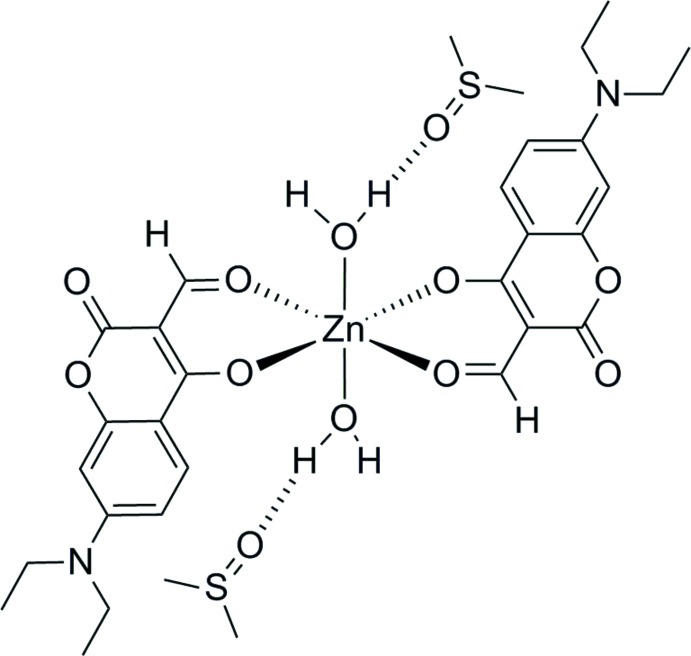


The detection of one particular metal ion, Zn^II^, is of special inter­est to our group. The Zn^II^ ion is ubiquitous in nature, playing important biological roles, and acting as a Lewis acid in the hydrolysis process involving carb­oxy­peptides. Zinc also plays many structural roles and is often found accompanied with cysteine and histidine residues (the classic zinc finger motif; Osredkar & Sustar, 2011 ▸). As a consequence of the filled *d* shell with its *d* ^10^ electron configuration, the zinc ion is found in all geometrical arrangements, with the tetra­hedral and octa­hedral being the two most common motifs. Additionally Zn^II^ is spectroscopically silent, therefore direct monitoring of this ion is challenging, especially in aqueous media. Our intention was to synthesize a planar mol­ecular chemosensor with a high degree of conjugation which can be easily perturbed to produce a spectroscopic response upon the coordination of Zn^II^ ions. In this paper we report the synthesis and the supra­molecular architecture of [Zn(7-di­ethyl­amino-3-formyl-chromen-2,4-dione)_2_(H_2_O)_2_], (1).

## Structural commentary   

**Figure 1:**
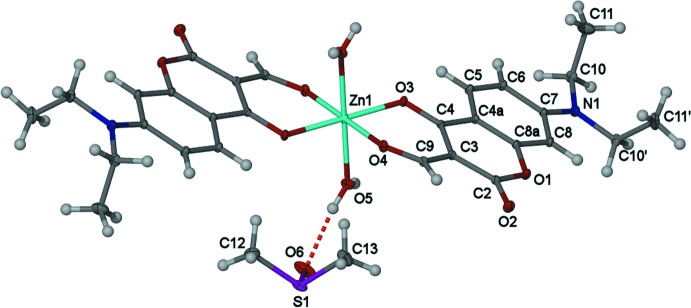
The mol­ecular structure of the title compound, showing displacement ellipsoids at the 50% probability level, with a single DMSO mol­ecule hydrogen bonded to a water mol­ecule coordinating to the zinc cation.

The mol­ecular structure of (1) is shown in Fig. 1 ▸. The coumarin ligand is planar and is coordinated to the Zn^II^ ion in a chelating fashion by the two carbonyl functional groups that form a pseudo-β-diketone motif. This is indicated by the short C=O bond of the dione (O3—C4) and the C=O bond length of the formyl moiety (O4—C9), with values of 1.2686 (10) and 1.2603 (10) Å, respectively. The Zn—O bonds complete the stable six-membered chelating motif, which is favorable for smaller metal ions (Hancock & Martell, 1989 ▸). The lengths of the Zn—O (carbon­yl) bond Zn1—O3 [2.0221 (6) Å] and the Zn—O (form­yl) bond Zn1—O4 [2.063 (6) Å] in the equatorial positions are in excellent agreement with similar chelating motifs (Dong *et al.*, 2010 ▸). The metal ion is located on an inversion center. The axial positions are occupied with two water mol­ecules, the Zn1—O5 bond length is at 2.1624 (7) Å slightly longer than that in other hydrated Zn^II^ coordination complexes, whereby the average Zn—O (aqua ligand) distance is 2.09 Å (Nimmermark *et al.*, 2013 ▸). The coordination sphere of the Zn^II^ ion is a near perfect octa­hedron with all of the bond angles close to 90°, ranging from 86.82 (3) to 93.18 (3)°. A single DMSO solvent mol­ecule completes the asymmetric unit.

## Supra­molecular features   

**Figure 2:**
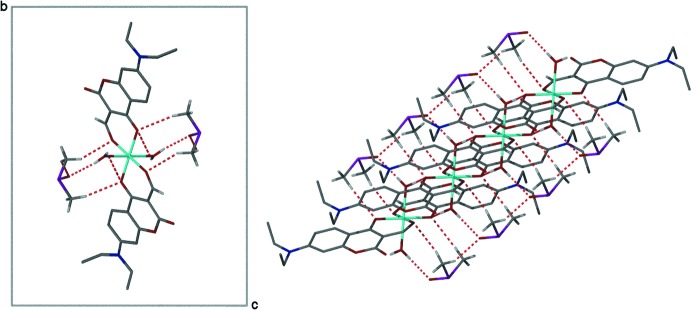
The crystal packing of the title compound highlighting the extensive hydrogen-bond network. The left side is the view down [100] and the right view highlights the five unique hydrogen-bonding inter­actions and three 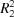
(8) systems.

The crystal structure of the title compound shows an extensive array of hydrogen-bonding inter­actions (Table 1 ▸) forming hydrogen-bond ring systems and infinite chains (Fig. 2 ▸). The encapsulated DMSO solvent mol­ecule forms a hydrogen-bonding inter­action with a single water mol­ecule that is coordinating to the Zn^II^ ion S1—O6⋯H52—O5 [1.983 (9) Å]. Inter­estingly, there are also two C—H⋯O hydrogen-bonding inter­actions from the methyl moiety of DMSO; one with the O atom on the formyl functional group in the equatorial position (H13*A*⋯O4 = 2.52 Å) and an additional hydrogen-bonding inter­action from the carbonyl­dione group occupying another equatorial position (H12*B*⋯O3 = 2.62 Å). Together these two inter­actions form three 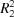
(8) systems. Furthermore, the DMSO solvent mol­ecule encapsulated within the crystal structure forms a single hydrogen-bonding inter­action with an adjacent DMSO mol­ecule H13*C*⋯O6(*x* + 1, *y*, *z*) (2.29 Å), forming an infinite chain.

**Figure 3:**
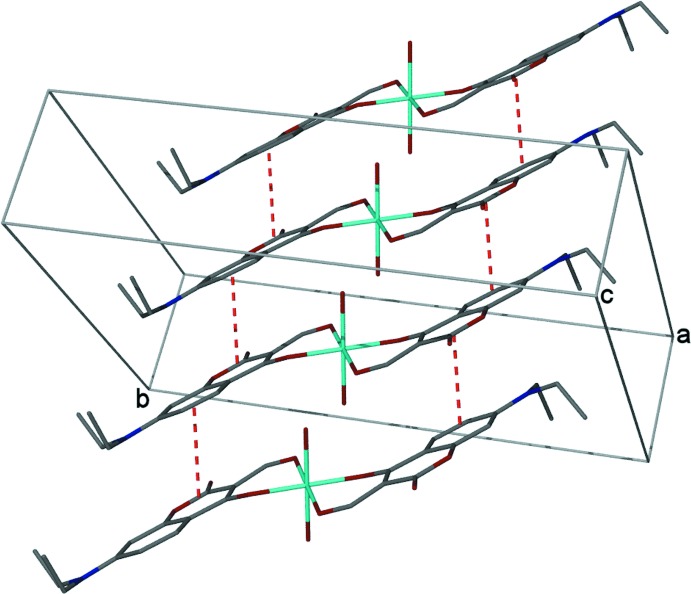
Side view of the crystal packing showing both the unit cell and the π–π stacking (3.734 Å). DMSO mol­ecules have been removed for clarity.

**Figure 4:**
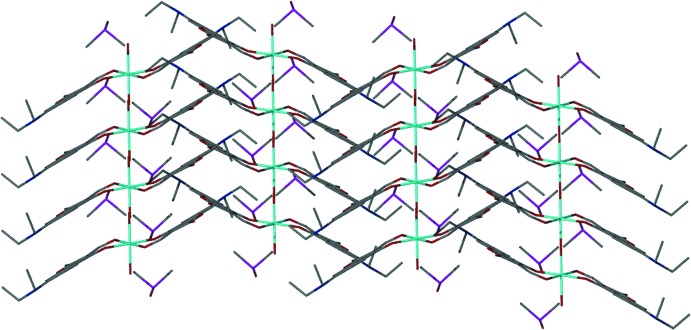
Side view of the crystal packing showing the π–π stacking of the coumarin of adjacent coordination complexes, emphasizing the zigzag motif.

It is well known that coumarin crystal packing displays π-stacking motifs as a consequence of the planarity of the organic framework (Guha *et al.*, 2013 ▸). Inter­estingly, the crystal packing of the title compound is influenced by off-set π–π inter­actions between the electron deficient coumarin ring system of one mol­ecule (ring system O1–C8*A*) and the electron-rich region of the second coumarin ring system (C4*A*–C8*A*) of an adjacent compound, whereby the centroids are 3.734 Å apart (Fig. 3 ▸). This is in good agreement with other π-stacking motifs (Wallace *et al.*, 2005 ▸). As a consequence, the packing arrangement shows a distinct zigzag pattern (Fig. 4 ▸).

## Database survey   

**Figure 5:**
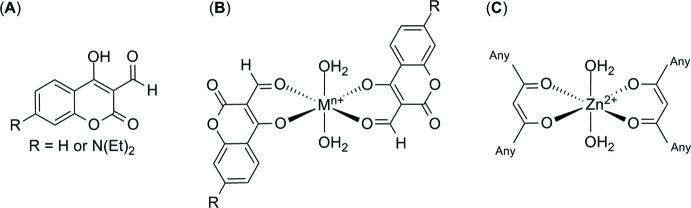
Chemical structures used in the CSD similarity search.

For coumarin-derived mol­ecular probes for the detection of neutral compounds, see: Wallace *et al.* (2006 ▸). A coumarin-based chemosensor for the detection of copper(II) ions was prepared by Xu *et al.* (2015 ▸). There are very few literature examples of Michael acceptors with cyanide that have been isolated, however Sun *et al.* (2012 ▸) have published an elegant crystal structure of a coumarin-cyanide adduct. There are over 25,000 zinc(II) coordination complexes in the Cambridge Structure Database (CSD; Groom *et al.*, 2016 ▸), both the tetra­hedral and octa­hedral environments. Therefore, the authors carried out a refined structure search based on the structures shown in Figs. 5 ▸(*a*) and 5(*b*); however, these did not yield any results. Therefore a modification of the search by specifically searching structures that have a bidentate chelating β-diketone motif coordinated to the zinc(II) in the equatorial position, with two water mol­ecules in the axial position, as shown in Fig. 5 ▸(*c*) was carried out. This refined search yielded two similar structures with Zn^II^ octahedrally coordinated, the first by Solans *et al.*, whereby two 1,3-bis­(2-hy­droxy­phen­yl)propane-1,3-dionate ligands coordinate to the Zn^II^ ion, with the remaining two coordination sites occupied by two ethanol mol­ecules (Solans *et al.*, 1983 ▸). The other similar structure was reported by Dong *et al.* (2010 ▸) who incorporated two 2-(4-benzo­yloxy-2-hy­droxy­benzo­yl)-1-phenyl­ethenolate ligands that were bound to the metal ion in the equatorial position and two ethanol mol­ecules situated in the axial postions.

## Synthesis and crystallization   

7-(Di­ethyl­amino)-4-hy­droxy­coumarin (467 mg, 2.00 mmol) was dissolved in 2-propanol (20 mL), tri­ethyl ­orthoformate (500 µL, 3.00 mmol) and 2-amino­pyriimidine (190 mg, 2.00 mmol) were added and the solution was heated to reflux for 4 h. Upon cooling, the solid was collected and used without further purification. This compound (200 mg, 0.59 mmol) was then dissolved in methanol (10 mL), to which Zn(OAc)_2_ (130 mg, 0.59 mmol) was then added to the solution. After stirring for 20 min, a yellow solid formed, which was collected by filtration and dried. A small amount of the solid (20 mg) was redissolved in a 1:1 mixture of MeOH and DMSO to form a saturated solution (1 mL) which was was allowed to stand for several weeks to form the title compound as colorless needles suitable for X-ray analysis. ^1^H NMR (300 K, CHCl_3_-*d*, 600 MHz p.p.m.): δ 9.68 (*s*, 2H, CHO), 7.91 (*d*, 2H, *J* = 2.4 Hz, ArH), 6.53 (*d*, *J* = 2.3 Hz, ArH), 6.33 (*s*, 2H, ArH), 3.41 (*q*, 8H, *J* = 7.1 Hz, CH_2_), 1.23 (*t*, 12H, *J* = 7.1 Hz, CH_3_); ^13^C NMR (300 K, CHCl_3_-*d*, 150 MHz p.p.m.) δ 192.2, 169.1, 165.8, 159.5, 157.7, 153.3, 128.3, 108.4, 108.0, 102.8, 96.9, 44.9, 40.6, 29.7, 12.5; LRMS–ESI (negative mode), NaCl was added as a charging agent [*M* − 2H_2_O + Cl]^−^ = 619 *m*/*z*, [*M* − H_2_O − C_14_H_15_NO_4_ + 2Cl]^−^ = 396 *m*/*z*, CID 396 yields [C_14_H_15_NO_4_]^−^ = 261 *m*/*z*; IR (ATR solid); 3364 (*br*, *s*) ν_OH_, 2972, 2926 (*m*) ν_CH_, 1722 (*m*) ν_CO_ (δ-lactone), 1689 ν_CO_ (ketone), 1590 ν_CO_ (form­yl), 564 ν_CO_ (Zn—O) cm^−1^.

## Refinement   

Crystal data, data collection and structure refinement details are summarized in Table 2 ▸. H atoms on C were idealized with a C—H distance of 0.95 Å for C*sp* ^2^, 0.99 Å for CH_2_, and 0.98 Å for methyl groups. Those on O atoms were assigned from difference maps, and their positions refined, with O—H distances restrained to 0.86 (1) Å. *U*_iso_ values for H atoms were assigned as 1.2 times *U*_eq_ of the attached atoms (1.5 for methyl and water groups).

## Supplementary Material

Crystal structure: contains datablock(s) I. DOI: 10.1107/S2056989016009853/zl2668sup1.cif

Structure factors: contains datablock(s) I. DOI: 10.1107/S2056989016009853/zl2668Isup2.hkl

CCDC reference: 1486125

Additional supporting information: crystallographic information; 3D view; checkCIF report

## Figures and Tables

**Table 1:** Hydrogen-bond geometry (Å, °)

*D*—H⋯*A*	*D*—H	H⋯*A*	*D*⋯*A*	*D*—H⋯*A*
O5—H52⋯O6	0.83 (1)	1.98 (1)	2.8030 (11)	171 (2)
O5—H51⋯O4^i^	0.83 (1)	1.99 (1)	2.8126 (9)	169 (1)
C12—H12*B*⋯O3^ii^	0.98	2.62	3.5805 (12)	167
C13—H13*A*⋯O4	0.98	2.52	3.4050 (13)	151
C13—H13*C*⋯O6^iii^	0.98	2.29	3.1299 (14)	143

Symmetry codes: (i) 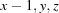; (ii) 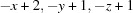; (iii) 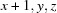.

**Table 2:** Experimental details

Crystal data	
Chemical formula	[Zn(C_14_H_14_NO_4_)_2_(H_2_O)_2_]·2C_2_H_6_OS
*M*_r_	778.18
Crystal system, space group	Monoclinic, *P*2_1_/*n*
Temperature (K)	90
*a*, *b*, *c* (Å)	5.2704 (2), 20.2885 (8), 16.0314 (8)
β (°)	94.210 (2)
*V* (Å^3^)	1709.59 (13)
*Z*	2
Radiation type	Mo *K*α
μ (mm^−1^)	0.91
Crystal size (mm)	0.42 × 0.13 × 0.06

Data collection	
Diffractometer	Bruker Kappa APEXII CCD DUO
Absorption correction	Multi-scan (*SADABS*; Sheldrick, 2004 ▸)
*T*_min_, *T*_max_	0.839, 0.948
No. of measured, independent and observed [*I* > 2σ(*I*)] reflections	52833, 7923, 6800
*R*_int_	0.034
(sin θ/λ)_max_ (Å^−1^)	0.821

Refinement	
*R*[*F* ^2^ > 2σ(*F* ^2^)], *wR*(*F* ^2^), *S*	0.029, 0.074, 1.05
No. of reflections	7923
No. of parameters	233
No. of restraints	2
H-atom treatment	H atoms treated by a mixture of independent and constrained refinement
Δρ_max_, Δρ_min_ (e Å^−3^)	0.64, −0.29

Computer programs: *APEX2* and *SAINT* (Bruker, 2009 ▸), *SHELXS97* (Sheldrick, 2008 ▸) and *SHELXL2014* (Sheldrick, 2015 ▸).

## supplementary crystallographic information

### Crystal data

**Table:**

[Zn(C_14_H_14_NO_4_)_2_(H_2_O)_2_]·2C_2_H_6_OS	*F*(000) = 816
*M**_r_* = 778.18	*D*_x_ = 1.512 Mg m^−^^3^
Monoclinic, *P*2_1_/*n*	Mo *K*α radiation, λ = 0.71073 Å
*a* = 5.2704 (2) Å	Cell parameters from 9942 reflections
*b* = 20.2885 (8) Å	θ = 2.7–35.6°
*c* = 16.0314 (8) Å	µ = 0.91 mm^−^^1^
β = 94.210 (2)°	*T* = 90 K
*V* = 1709.59 (13) Å^3^	Needle, colorless
*Z* = 2	0.42 × 0.13 × 0.06 mm

### Data collection

**Table:**

Bruker Kappa APEXII CCD DUO diffractometer	7923 independent reflections
Radiation source: fine-focus sealed tube	6800 reflections with *I* > 2σ(*I*)
TRIUMPH curved graphite monochromator	*R*_int_ = 0.034
φ and ω scans	θ_max_ = 35.7°, θ_min_ = 1.6°
Absorption correction: multi-scan (SADABS; Sheldrick, 2004)	*h* = −8→8
*T*_min_ = 0.839, *T*_max_ = 0.948	*k* = −32→33
52833 measured reflections	*l* = −26→26

### Refinement

**Table:**

Refinement on *F*^2^	2 restraints
Least-squares matrix: full	Hydrogen site location: mixed
*R*[*F*^2^ > 2σ(*F*^2^)] = 0.029	H atoms treated by a mixture of independent and constrained refinement
*wR*(*F*^2^) = 0.074	*w* = 1/[σ^2^(*F*_o_^2^) + (0.037*P*)^2^ + 0.4839*P*] where *P* = (*F*_o_^2^ + 2*F*_c_^2^)/3
*S* = 1.05	(Δ/σ)_max_ = 0.001
7923 reflections	Δρ_max_ = 0.64 e Å^−^^3^
233 parameters	Δρ_min_ = −0.29 e Å^−^^3^

### Special details

**Table:**

Geometry. All esds (except the esd in the dihedral angle between two l.s. planes) are estimated using the full covariance matrix. The cell esds are taken into account individually in the estimation of esds in distances, angles and torsion angles; correlations between esds in cell parameters are only used when they are defined by crystal symmetry. An approximate (isotropic) treatment of cell esds is used for estimating esds involving l.s. planes.

### Fractional atomic coordinates and isotropic or equivalent isotropic displacement parameters (Å^2^)

**Table:**

	*x*	*y*	*z*	*U*_iso_*/*U*_eq_	
Zn1	1.0000	0.5000	0.5000	0.00796 (3)	
O1	0.76292 (12)	0.75420 (3)	0.39084 (4)	0.01077 (11)	
O2	1.07150 (13)	0.72578 (3)	0.31308 (4)	0.01409 (12)	
O3	0.84598 (12)	0.58615 (3)	0.53466 (4)	0.00965 (10)	
O4	1.23910 (12)	0.55264 (3)	0.42845 (4)	0.01070 (11)	
O5	0.71499 (13)	0.49969 (3)	0.39585 (4)	0.01339 (12)	
H51	0.5673 (19)	0.5100 (7)	0.4057 (10)	0.020*	
H52	0.697 (3)	0.4765 (7)	0.3534 (7)	0.020*	
N1	0.08901 (14)	0.83166 (4)	0.54592 (5)	0.01113 (12)	
C2	0.95013 (16)	0.71005 (4)	0.37136 (5)	0.00949 (13)	
C3	0.97983 (15)	0.64990 (4)	0.42057 (5)	0.00849 (12)	
C4	0.82637 (15)	0.63719 (4)	0.48897 (5)	0.00782 (12)	
C4A	0.64144 (15)	0.68716 (4)	0.50673 (5)	0.00800 (12)	
C5	0.48155 (16)	0.68183 (4)	0.57310 (5)	0.00925 (13)	
H5	0.4947	0.6441	0.6082	0.011*	
C6	0.30707 (16)	0.72985 (4)	0.58833 (5)	0.01032 (13)	
H6	0.2068	0.7257	0.6349	0.012*	
C7	0.27487 (16)	0.78592 (4)	0.53494 (5)	0.00915 (13)	
C8	0.43691 (16)	0.79177 (4)	0.46922 (5)	0.00975 (13)	
H8	0.4241	0.8291	0.4335	0.012*	
C8A	0.61446 (16)	0.74331 (4)	0.45661 (5)	0.00859 (12)	
C9	1.18006 (16)	0.60867 (4)	0.39914 (5)	0.00982 (13)	
H9	1.2830	0.6251	0.3576	0.012*	
C10	−0.05833 (17)	0.83018 (5)	0.61973 (6)	0.01397 (15)	
H10A	−0.1134	0.7843	0.6292	0.017*	
H10B	−0.2132	0.8573	0.6086	0.017*	
C11	0.0875 (2)	0.85532 (6)	0.69888 (6)	0.0237 (2)	
H11A	0.2527	0.8331	0.7061	0.036*	
H11B	−0.0103	0.8461	0.7473	0.036*	
H11C	0.1138	0.9030	0.6943	0.036*	
C10'	0.05448 (17)	0.88743 (4)	0.48901 (6)	0.01302 (14)	
H10C	−0.1202	0.9048	0.4918	0.016*	
H10D	0.0711	0.8718	0.4312	0.016*	
C11'	0.24407 (19)	0.94345 (5)	0.50790 (7)	0.01896 (18)	
H11D	0.2154	0.9628	0.5624	0.028*	
H11E	0.2204	0.9773	0.4644	0.028*	
H11F	0.4179	0.9261	0.5089	0.028*	
S1	0.92308 (4)	0.42229 (2)	0.19940 (2)	0.01629 (5)	
O6	0.68156 (15)	0.43188 (5)	0.24310 (6)	0.0304 (2)	
C12	1.0926 (2)	0.35719 (6)	0.25330 (6)	0.02078 (19)	
H12A	1.0056	0.3153	0.2408	0.031*	
H12B	1.0995	0.3654	0.3137	0.031*	
H12C	1.2659	0.3551	0.2351	0.031*	
C13	1.1280 (2)	0.48866 (6)	0.23282 (8)	0.0241 (2)	
H13A	1.1447	0.4902	0.2941	0.036*	
H13B	1.0559	0.5303	0.2110	0.036*	
H13C	1.2959	0.4819	0.2117	0.036*	

### Atomic displacement parameters (Å^2^)

**Table:**

	*U*^11^	*U*^22^	*U*^33^	*U*^12^	*U*^13^	*U*^23^
Zn1	0.00668 (6)	0.00682 (6)	0.01065 (6)	0.00185 (4)	0.00248 (4)	−0.00008 (4)
O1	0.0130 (3)	0.0096 (3)	0.0103 (2)	0.0026 (2)	0.0048 (2)	0.00221 (19)
O2	0.0158 (3)	0.0148 (3)	0.0124 (3)	0.0006 (2)	0.0065 (2)	0.0027 (2)
O3	0.0115 (3)	0.0071 (2)	0.0107 (2)	0.0026 (2)	0.00331 (19)	0.00131 (18)
O4	0.0085 (2)	0.0094 (2)	0.0147 (3)	0.0012 (2)	0.0038 (2)	−0.0001 (2)
O5	0.0082 (3)	0.0180 (3)	0.0140 (3)	0.0031 (2)	0.0009 (2)	−0.0041 (2)
N1	0.0107 (3)	0.0101 (3)	0.0127 (3)	0.0043 (2)	0.0020 (2)	0.0003 (2)
C2	0.0098 (3)	0.0096 (3)	0.0092 (3)	0.0001 (3)	0.0018 (2)	−0.0003 (2)
C3	0.0086 (3)	0.0084 (3)	0.0088 (3)	0.0007 (2)	0.0028 (2)	0.0002 (2)
C4	0.0074 (3)	0.0077 (3)	0.0084 (3)	0.0004 (2)	0.0008 (2)	−0.0006 (2)
C4A	0.0079 (3)	0.0073 (3)	0.0090 (3)	0.0012 (2)	0.0020 (2)	0.0001 (2)
C5	0.0094 (3)	0.0088 (3)	0.0097 (3)	0.0015 (2)	0.0024 (2)	0.0014 (2)
C6	0.0105 (3)	0.0095 (3)	0.0113 (3)	0.0024 (3)	0.0031 (3)	0.0013 (2)
C7	0.0088 (3)	0.0081 (3)	0.0105 (3)	0.0017 (2)	0.0006 (2)	−0.0009 (2)
C8	0.0110 (3)	0.0077 (3)	0.0107 (3)	0.0023 (3)	0.0017 (2)	0.0010 (2)
C8A	0.0094 (3)	0.0079 (3)	0.0086 (3)	0.0005 (2)	0.0019 (2)	0.0005 (2)
C9	0.0086 (3)	0.0102 (3)	0.0110 (3)	−0.0003 (3)	0.0031 (2)	−0.0008 (2)
C10	0.0112 (3)	0.0149 (4)	0.0163 (4)	0.0037 (3)	0.0041 (3)	−0.0010 (3)
C11	0.0239 (5)	0.0321 (6)	0.0155 (4)	0.0050 (4)	0.0034 (3)	−0.0067 (4)
C10'	0.0106 (3)	0.0103 (3)	0.0180 (4)	0.0031 (3)	0.0001 (3)	0.0014 (3)
C11'	0.0153 (4)	0.0106 (4)	0.0312 (5)	0.0008 (3)	0.0028 (4)	−0.0005 (3)
S1	0.00995 (9)	0.02305 (11)	0.01552 (9)	0.00354 (8)	−0.00148 (7)	−0.00841 (8)
O6	0.0089 (3)	0.0480 (5)	0.0346 (4)	0.0016 (3)	0.0025 (3)	−0.0249 (4)
C12	0.0204 (4)	0.0264 (5)	0.0155 (4)	0.0024 (4)	0.0017 (3)	0.0002 (3)
C13	0.0160 (4)	0.0238 (5)	0.0319 (5)	0.0009 (4)	−0.0015 (4)	−0.0105 (4)

### Geometric parameters (Å, º)

**Table:**

Zn1—O3^i^	2.0221 (6)	C7—C8	1.4090 (11)
Zn1—O3	2.0221 (6)	C8—C8A	1.3823 (11)
Zn1—O4	2.0631 (6)	C8—H8	0.9500
Zn1—O4^i^	2.0632 (6)	C9—H9	0.9500
Zn1—O5	2.1624 (7)	C10—C11	1.5224 (14)
Zn1—O5^i^	2.1624 (7)	C10—H10A	0.9900
O1—C8A	1.3762 (10)	C10—H10B	0.9900
O1—C2	1.3852 (10)	C11—H11A	0.9800
O2—C2	1.2132 (10)	C11—H11B	0.9800
O3—C4	1.2683 (10)	C11—H11C	0.9800
O4—C9	1.2603 (10)	C10'—C11'	1.5289 (14)
O5—H51	0.832 (9)	C10'—H10C	0.9900
O5—H52	0.827 (9)	C10'—H10D	0.9900
N1—C7	1.3700 (11)	C11'—H11D	0.9800
N1—C10'	1.4565 (11)	C11'—H11E	0.9800
N1—C10	1.4624 (12)	C11'—H11F	0.9800
C2—C3	1.4552 (11)	S1—O6	1.5100 (8)
C3—C9	1.4088 (11)	S1—C12	1.7822 (11)
C3—C4	1.4330 (11)	S1—C13	1.7836 (11)
C4—C4A	1.4490 (11)	C12—H12A	0.9800
C4A—C8A	1.3953 (11)	C12—H12B	0.9800
C4A—C5	1.4091 (11)	C12—H12C	0.9800
C5—C6	1.3739 (11)	C13—H13A	0.9800
C5—H5	0.9500	C13—H13B	0.9800
C6—C7	1.4263 (12)	C13—H13C	0.9800
C6—H6	0.9500		
			
O3^i^—Zn1—O3	180.00 (3)	C7—C8—H8	119.9
O3^i^—Zn1—O4	91.19 (2)	O1—C8A—C8	115.27 (7)
O3—Zn1—O4	88.81 (2)	O1—C8A—C4A	122.18 (7)
O3^i^—Zn1—O4^i^	88.81 (2)	C8—C8A—C4A	122.55 (7)
O3—Zn1—O4^i^	91.19 (2)	O4—C9—C3	127.84 (8)
O4—Zn1—O4^i^	180.0	O4—C9—H9	116.1
O3^i^—Zn1—O5	93.18 (3)	C3—C9—H9	116.1
O3—Zn1—O5	86.82 (3)	N1—C10—C11	113.70 (8)
O4—Zn1—O5	89.44 (3)	N1—C10—H10A	108.8
O4^i^—Zn1—O5	90.56 (3)	C11—C10—H10A	108.8
O3^i^—Zn1—O5^i^	86.83 (3)	N1—C10—H10B	108.8
O3—Zn1—O5^i^	93.17 (3)	C11—C10—H10B	108.8
O4—Zn1—O5^i^	90.56 (3)	H10A—C10—H10B	107.7
O4^i^—Zn1—O5^i^	89.44 (3)	C10—C11—H11A	109.5
O5—Zn1—O5^i^	180.00 (4)	C10—C11—H11B	109.5
C8A—O1—C2	121.56 (6)	H11A—C11—H11B	109.5
C4—O3—Zn1	124.36 (5)	C10—C11—H11C	109.5
C9—O4—Zn1	122.01 (5)	H11A—C11—H11C	109.5
Zn1—O5—H51	117.4 (11)	H11B—C11—H11C	109.5
Zn1—O5—H52	131.9 (11)	N1—C10'—C11'	113.80 (8)
H51—O5—H52	104.3 (15)	N1—C10'—H10C	108.8
C7—N1—C10'	120.21 (7)	C11'—C10'—H10C	108.8
C7—N1—C10	121.15 (7)	N1—C10'—H10D	108.8
C10'—N1—C10	118.26 (7)	C11'—C10'—H10D	108.8
O2—C2—O1	115.34 (7)	H10C—C10'—H10D	107.7
O2—C2—C3	126.61 (8)	C10'—C11'—H11D	109.5
O1—C2—C3	118.04 (7)	C10'—C11'—H11E	109.5
C9—C3—C4	123.69 (7)	H11D—C11'—H11E	109.5
C9—C3—C2	114.74 (7)	C10'—C11'—H11F	109.5
C4—C3—C2	121.43 (7)	H11D—C11'—H11F	109.5
O3—C4—C3	124.22 (7)	H11E—C11'—H11F	109.5
O3—C4—C4A	119.03 (7)	O6—S1—C12	106.27 (6)
C3—C4—C4A	116.75 (7)	O6—S1—C13	106.01 (5)
C8A—C4A—C5	117.14 (7)	C12—S1—C13	98.22 (6)
C8A—C4A—C4	119.98 (7)	S1—C12—H12A	109.5
C5—C4A—C4	122.88 (7)	S1—C12—H12B	109.5
C6—C5—C4A	121.67 (7)	H12A—C12—H12B	109.5
C6—C5—H5	119.2	S1—C12—H12C	109.5
C4A—C5—H5	119.2	H12A—C12—H12C	109.5
C5—C6—C7	120.71 (7)	H12B—C12—H12C	109.5
C5—C6—H6	119.6	S1—C13—H13A	109.5
C7—C6—H6	119.6	S1—C13—H13B	109.5
N1—C7—C8	121.16 (7)	H13A—C13—H13B	109.5
N1—C7—C6	121.18 (7)	S1—C13—H13C	109.5
C8—C7—C6	117.64 (7)	H13A—C13—H13C	109.5
C8A—C8—C7	120.23 (7)	H13B—C13—H13C	109.5
C8A—C8—H8	119.9		
			
C8A—O1—C2—O2	−178.59 (8)	C10'—N1—C7—C6	−178.06 (8)
C8A—O1—C2—C3	2.40 (11)	C10—N1—C7—C6	9.15 (12)
O2—C2—C3—C9	3.39 (13)	C5—C6—C7—N1	175.22 (8)
O1—C2—C3—C9	−177.73 (7)	C5—C6—C7—C8	−3.26 (12)
O2—C2—C3—C4	179.19 (8)	N1—C7—C8—C8A	−176.64 (8)
O1—C2—C3—C4	−1.93 (11)	C6—C7—C8—C8A	1.84 (12)
Zn1—O3—C4—C3	−22.08 (11)	C2—O1—C8A—C8	179.44 (7)
Zn1—O3—C4—C4A	159.09 (6)	C2—O1—C8A—C4A	−0.73 (12)
C9—C3—C4—O3	−3.62 (13)	C7—C8—C8A—O1	−179.82 (7)
C2—C3—C4—O3	−179.04 (8)	C7—C8—C8A—C4A	0.35 (13)
C9—C3—C4—C4A	175.24 (7)	C5—C4A—C8A—O1	179.02 (7)
C2—C3—C4—C4A	−0.18 (11)	C4—C4A—C8A—O1	−1.52 (12)
O3—C4—C4A—C8A	−179.20 (7)	C5—C4A—C8A—C8	−1.16 (12)
C3—C4—C4A—C8A	1.88 (11)	C4—C4A—C8A—C8	178.30 (8)
O3—C4—C4A—C5	0.24 (12)	Zn1—O4—C9—C3	13.02 (12)
C3—C4—C4A—C5	−178.68 (7)	C4—C3—C9—O4	8.25 (14)
C8A—C4A—C5—C6	−0.29 (12)	C2—C3—C9—O4	−176.05 (8)
C4—C4A—C5—C6	−179.74 (8)	C7—N1—C10—C11	75.54 (11)
C4A—C5—C6—C7	2.53 (13)	C10'—N1—C10—C11	−97.39 (10)
C10'—N1—C7—C8	0.37 (12)	C7—N1—C10'—C11'	−80.01 (10)
C10—N1—C7—C8	−172.43 (8)	C10—N1—C10'—C11'	92.99 (10)

Symmetry code: (i) −*x*+2, −*y*+1, −*z*+1.

### Hydrogen-bond geometry (Å, º)

**Table:**

*D*—H···*A*	*D*—H	H···*A*	*D*···*A*	*D*—H···*A*
O5—H52···O6	0.83 (1)	1.98 (1)	2.8030 (11)	171 (2)
O5—H51···O4^ii^	0.83 (1)	1.99 (1)	2.8126 (9)	169 (1)
C12—H12*B*···O3^i^	0.98	2.62	3.5805 (12)	167
C13—H13*A*···O4	0.98	2.52	3.4050 (13)	151
C13—H13*C*···O6^iii^	0.98	2.29	3.1299 (14)	143

Symmetry codes: (i) −*x*+2, −*y*+1, −*z*+1; (ii) *x*−1, *y*, *z*; (iii) *x*+1, *y*, *z*.
